# Effect of kernel breakage on the fermentation profile, nitrogen fractions, and in vitro starch digestibility of whole-plant corn silage and ensiled corn grain

**DOI:** 10.3168/jdsc.2021-0093

**Published:** 2021-06-23

**Authors:** B.A. Saylor, E.C. Diepersloot, C. Heinzen, C.L. McCary, L.F. Ferraretto

**Affiliations:** 1 Department of Animal and Dairy Sciences, University of Wisconsin, Madison 53706; 2 Department of Animal Sciences, University of Florida, Gainesville 32608

## Abstract

The objective of this experiment was to analyze the effect of kernel breakage on the fermentation profile, nitrogen fractions, and ruminal in vitro starch digestibility of whole-plant corn silage and ensiled corn grain. Whole corn plants were harvested, and ears were separated from the forage portion and shelled. Corn kernels were either left intact or broken manually using a hammer. The remaining forage portion of the corn plants was chopped. Samples of the intact and broken kernels were stored for 0 or 30 d in quadruplicate vacuum pouches. Remaining intact and broken kernels were each reconstituted with the chopped forage portion of the corn plant to simulate whole-plant corn forage and were also stored for 0 or 30 d. In kernels separated from whole-plant corn silage, kernel form had no effect on zein protein concentrations. However, it was observed that in vitro starch digestibility at 7 h increased with ensiling only in kernels that were broken. When corn kernels were ensiled alone, concentrations of soluble crude protein and ammonia nitrogen increased with ensiling to a greater extent when kernels were broken. Finally, fermentation of ensiled corn grain was enhanced when kernels were broken. Overall, this study gives insight into the importance of kernel breakage to improve starch digestibility in corn silage through means other than a reduction in particle size and opens the door for continued investigation into the proteolytic activity occurring in the silo.

## Highlights

• Fermentation was unaffected by kernel breakage in whole-plant corn silage.
• Starch digestibility increased with ensiling in broken but not intact kernels.
• Nitrogen fractions increased to a greater extent in broken kernels ensiled alone.
• Fermentation of ensiled corn grain was enhanced in broken kernels ensiled alone.

## Graphical Abstract

**Figure:**
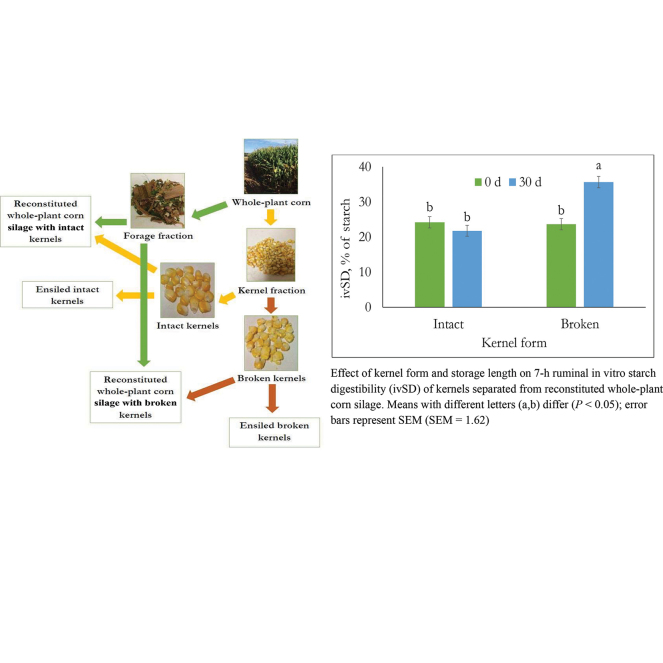
**Summary:** In this experiment, whole corn plants were harvested, and ears were separated from the forage portion and shelled. Corn kernels were either left intact or broken manually using a hammer. The remaining forage portion of the corn plants was chopped. Samples of the intact and broken kernels were stored for 0 or 30 d in quadruplicate vacuum pouches. Remaining intact and broken kernels were each reconstituted with the chopped forage portion of the corn plant to simulate whole-plant corn forage and were also stored for 0 or 30 d. Kernels separated from reconstituted whole-plant corn silage were analyzed for 7-h ruminal in vitro starch digestibility. It was observed that 7-h ruminal in vitro starch digestibility increased with ensiling only in kernels that were broken.

Starch from corn grain and silage is a common source of energy in the diets of high-producing dairy cows. Ensiling corn grain as whole-plant corn silage (**WPCS**) or high-moisture corn (**HMC**) is a widespread storage method for dairy farmers and increases ruminal and total-tract starch digestibility compared with dry ground corn (Ferraretto et al., 2013). During ensiling, the hydrophobic starch–protein matrices that surround starch granules are broken down, making starch more accessible for degradation by ruminal microorganisms (Kotarski et al., 1992). This response is intensified with prolonged storage (Kung et al., 2018).

Several mechanisms are responsible for this proteolytic activity in the silo, including kernel proteases, microorganisms, and solubilization by fermentation acids (Simpson, 2001; Junges et al., 2017). Junges et al. (2017) determined that microbial degradation was the primary proteolytic mechanism in corn grain silages, followed by corn kernel enzymes and organic acids. Mechanical processing of corn kernels breaks the pericarp and disrupts the starch–protein matrix, thereby facilitating this proteolytic activity and enhancing starch digestibility (Ferraretto et al., 2018). Saylor et al. (2020) reported greater ruminal in situ starch degradation for finely ground compared with coarsely ground HMC; this effect was related to a reduction in particle size and increased proteolysis during storage as indicated by greater soluble CP and ammonia N concentrations. These findings suggest that the degree of proteolysis is partially dependent on the physical form of ensiled kernels. Although it is well known that the presence of intact kernels in corn silage reduces starch digestibility (Ferraretto et al., 2018), the extent to which proteolysis occurs in whole kernels during ensiling requires further investigation. Therefore, the objective of this experiment was to analyze the effect of kernel breakage on the fermentation profile, N fractions, and ruminal in vitro starch digestibility at 7 h (**ivSD**) of WPCS and ensiled corn grain. We hypothesized that ensiling would increase starch digestibility in both WPCS and ensiled corn grain but that the effect would be greater when kernels were broken.

Whole corn plants (1024 VIP, AgraTech Seeds Inc.) at silage maturity (two-thirds kernel milkline) were harvested at the University of Florida Plant Science Research and Education Unit (Citra, FL) on October 18, 2018, from 4 random locations within the same field to correspond with 4 replicates per treatment. At harvest, ears were separated from the corn plants and shelled. Corn kernels were homogenized by location and left intact or broken manually using a hammer. Kernels were broken carefully, one at a time, to damage the pericarp without doing extensive damage to the physical integrity of the kernel. Kernels were still in one piece after being broken. The remaining forage portion of the corn plants was chopped with a single-row silage chopper (model no. 707 SN: 245797l; CNH Industrial America LLC). Samples of intact and broken kernels (200 g in size) were assigned to either 0 or 30 d of storage. Each combination of kernel form (intact or broken) and storage length (0 or 30 d) had 4 replicates (1 from each location). All samples (n = 16) were vacuum-sealed in nylon-polyethylene standard barrier vacuum pouches (0.09-mm thickness, 25.4 × 35.6 cm; Doug Care Equipment Inc.) using an external clamp vacuum machine (Bestvac).

Remaining intact and broken kernels were each reconstituted with the chopped forage portion of the corn plant to simulate whole-plant corn forage with starch concentrations of approximately 30%. Reconstituted whole-plant corn forage samples (500 g in size) were assigned to either 0 or 30 d of storage. Each combination of kernel form (intact or broken) and storage length (0 or 30 d) had 4 replicates (1 from each location). All whole-plant corn forage samples (n = 16) were ensiled using the same system as described above. All mini silos with a designated storage length of 0 d were sealed, weighed, and immediately frozen. All other mini silos were weighed and stored at room temperature (~20°C) in the dark for 30 d. After 30 d, all silos were opened. The material was homogenized, and one half of each sample was immediately frozen at −20°C to stop fermentation and stored until it could be processed for further analysis. Additionally, duplicate 50-g (as-fed) silage samples were dried at 60°C for 48 h in a forced-air oven (Heratherm OMS180, Thermo Fisher Scientific) to determine nonvolatile DM concentration and ground to pass through a 1-mm screen in a Wiley mill (A. H. Thomas Scientific).

At the time of silo opening, 20-g (as-fed) silage samples were mixed with 200 mL of 0.1% peptone water (Oxoid CM0090) in a stomacher machine (Lab-Blender 400, Tekmar Co.) for 1 min. The pH of the resulting extract was measured using a pH meter (Maizeing model 12, Maizeing Scientific Instruments). Approximately 40 mL of silage extract was filtered through 2 layers of cheesecloth into a 50-mL plastic centrifuge tube. Silage extract was then acidified with 0.4 mL of 50% sulfuric acid and centrifuged at 7,122 × *g* for 15 min at 4°C. The supernatant was analyzed for ammonia N using a Technicon Auto Analyzer (RFA-300, Alpkem Corp.) adapted from the Noel and Hambleton (1976) method for colorimetric ammonia quantification. A 2-mL subsample of the supernatant was collected and centrifuged again at 14,534 × *g* for 10 min at 4°C. The resulting supernatant was then filtered with a 0.22-µm syringe filter and used for quantification of organic acids using HPLC (Merck Hitachi Elite La-Chrome) with an isocratic elution system attached to an ion exclusion column (300 × 7.8-mm i.d.; Bio-Rad Aminex HPX-87H, Bio-Rad Laboratories) and a UV detector (Merck Hitachi L-2400) set at 210 nm with a 0.015 *M* sulfuric acid mobile phase and a flow rate of 0.7 mL/min at 45°C.

Reconstituted WPCS was analyzed for nutrient composition and fermentation profile at the University of Florida. Absolute DM was determined by oven-drying at 105°C for 3 h (method 2.2.2.5; NFTA, 1993). Starch concentrations were analyzed as described by Hall (2015). Concentrations of water-soluble carbohydrates (**WSC**) were determined by the anthrone reaction assay (Ministry of Agriculture, Fisheries, and Food, 1986). Total N was analyzed by the Dumas dry combustion method (method 968.06; AOAC International, 2016), with CP concentration calculated as N × 6.25. Borate phosphate-soluble CP was determined according to Krishnamoorthy et al. (1982).

Kernels separated from the reconstituted WPCS after the storage period were sent to Dairyland Laboratories Inc. (Arcadia, WI) for analysis of starch (Vidal et al., 2009), CP (method 990.03; AOAC International, 2016), borate phosphate-soluble CP (Krishnamoorthy et al., 1982), ammonia N (Peters et al., 2003, modified using Kjelsorb reagent), zein proteins (Larson and Hoffman, 2008), and ivSD (Richards et al., 1995). Ensiled corn grain samples were analyzed for DM, starch, WSC, ammonia N, pH, and organic acids at the University of Florida using methods described above. Concentrations of CP, soluble CP, and zein proteins as well as ivSD were analyzed by Dairyland Laboratories Inc. using the methods described above.

Data for the reconstituted WPCS, kernels separated from WPCS, and ensiled corn grain were analyzed separately. Data were analyzed as a completely randomized design using PROC GLIMMIX of SAS (SAS Institute Inc.). The model included kernel form, ensiling, and their interaction as fixed effects. Means were determined using the LSMEANS statement, and treatment means were compared using the Bonferroni *t*-test option after a significant overall treatment *F*-test. Statistical significance and trends were declared at *P* ≤ 0.05 and *P* > 0.05 to *P* ≤ 0.10, respectively.

Effects of kernel form and ensiling on the fermentation profile and nutrient composition of reconstituted WPCS are shown in Table 1. There tended to be an interaction between kernel form and ensiling for pH of WPCS (*P* = 0.09). Silage pH was reduced at 30 d of storage compared with at 0 d for silage containing intact and broken kernels. However, pH at 0 d of storage was greater in silage containing intact kernels compared with silage containing broken kernels. It is well understood that accumulation of lactic acid is the primary contributor to the decline in pH during the early stages of ensiling (Kung et al., 2018). The slightly greater pH in silage containing intact kernels at d 0 is within typical pH values for whole-plant corn forage immediately after chopping (5.5–6; Kung et al., 2018) and of minor biological significance. Concentrations of lactic, acetic, and total acids were unaffected (*P* > 0.10) by kernel form. As expected, concentrations of lactic, acetic, and total acids were greater (*P* < 0.001) at 30 d of storage compared with at 0 d.

**Table 1:** Effect (means) of kernel form and ensiling on the fermentation profile and nutrient composition of reconstituted whole-plant corn silage

Item	Kernel		Storage length		Intact		Broken		SEM	*P*-value^1^		
	Intact	Broken	0 d	30 d	0 d	30 d	0 d	30 d		K	E	K × E
pH	5.1	4.9	5.9	4.0	6.1	4.0	5.8	4.0	0.05	0.02	<0.001	0.09
Lactic acid, % of DM	2.9	2.5	0.03	5.4	0.03	5.7	0.03	5.1	0.24	0.17	<0.001	0.17
Acetic acid, % of DM	0.7	0.7	0.01	1.3	0.01	1.4	0.01	1.3	0.05	0.52	<0.001	0.53
Total acids, % of DM	4.0	3.6	0.5	7.1	0.5	7.5	0.4	6.7	0.28	0.18	<0.001	0.19
DM, % as-fed	37.5	37.8	38.0	37.3	37.5	37.5	38.6	37.1	1.21	0.79	0.54	0.55
Starch, % of DM	28.4	27.3	28.9	26.9	29.3	27.5	28.4	26.2	1.43	0.43	0.18	0.88
WSC,^2^ % of DM	5.1	4.9	8.3	1.7	8.5	1.7	8.1	1.7	0.42	0.62	<0.001	0.66
CP, % of DM	8.8	8.8	8.7	8.9	8.6	9.0	8.8	8.9	0.21	0.73	0.22	0.46
Soluble CP, % of CP	30.7	30.4	24.7	36.4	25.3	36.1	24.1	36.6	1.23	0.78	<0.001	0.53
Ammonia N, % of CP	19.7	19.7	10.8	28.6	12.3	27.2	9.3	30.1	1.73	0.99	<0.001	0.12

1 K = kernel effect (intact vs. broken); E = ensiling effect (0 vs. 30 d).

2 Water-soluble carbohydrates.

No effects (*P* > 0.10) of kernel form or ensiling were observed for concentrations of DM and starch. Ensiling reduced (*P* < 0.001) concentrations of WSC, which were presumably used as substrate for the growth and development of lactic acid-producing bacteria (Kung et al., 2018). Concentrations of CP, soluble CP, and ammonia N in WPCS were unaffected (*P* > 0.10) by kernel form. Soluble CP and ammonia N concentrations, but not CP, were affected (*P* < 0.001) by ensiling, with elevated concentrations of both N fractions in WPCS stored for 30 d compared with unfermented silage. It is well documented that substantial proteolysis occurs in WPCS during the first 30 d of storage, as evidenced by increased concentrations of ammonia N and soluble CP (Kung et al., 2018). Junges et al. (2017) reported that bacterial activity was the primary contributor to proteolysis (60%), followed by plant enzymes (30%), fungi (5%), and fermentation end products (5%).

The effect of kernel form and ensiling on the nutrient composition and N fractions of kernels separated from reconstituted WPCS is shown in Table 2. Concentrations of DM were unaffected by kernel form (*P* > 0.10), but ensiling tended (*P* = 0.09) to reduce DM concentrations by 1.3 percentage units. An interaction between kernel form and ensiling was observed (*P* = 0.01) for concentrations of CP. Crude protein concentrations were not different between d 0 and 30 in intact kernels, but in broken kernels, concentrations of CP were 1.0 percentage unit lower at d 30 than they were at d 0. An interaction between kernel form and ensiling was also observed for concentrations of soluble CP (*P* = 0.05). Soluble CP concentrations increased with storage in both intact and broken kernels but to a greater extent in kernels that were intact. Concentrations of zein proteins in kernels separated from reconstituted WPCS were unaffected by treatments (*P* > 0.10). Zein proteins are the primary proteins in the corn starch–protein matrix and can contribute to more than 50% of the total protein in corn grain (Hamaker et al., 1995). Our soluble CP and ammonia N data suggest greater proteolytic activity in intact kernels, which is unexpected. As described previously, breaking the pericarp of corn kernels and disrupting the starch–protein matrix through mechanical processing were hypothesized to increase proteolytic activity by bacteria. Why these N fractions were elevated in intact kernels is unclear. The corn endosperm contains various proteases responsible for the proteolysis of endosperm proteins during germination (Simpson, 2001). These proteases are most active at a pH between 4 and 5.5 (Simpson, 2001). It is possible that these kernel enzymes, rather than bacterial proteases, were responsible for the proteolysis that occurred in intact kernels.

**Table 2:** Effect (means) of kernel form and ensiling on the nutrient composition and N fractions of kernels separated from reconstituted whole-plant corn silage

Item	Kernel		Storage length		Intact		Broken		SEM	*P*-value^1^		
	Intact	Broken	0 d	30 d	0 d	30 d	0 d	30 d		K	E	K × E
DM, % as-fed	60.1	59.1	60.3	59.0	60.9	59.3	59.7	58.6	0.70	0.19	0.09	0.75
CP, % of DM	10.1	9.5	10.1	9.5	10.2^a^	10.0^a^	10.0^a^	9.0^b^	0.12	<0.001	<0.001	0.01
Soluble CP, % of CP	20.4	18.2	15.3	23.2	15.7^c^	25.1^a^	15.0^c^	21.3^b^	0.74	0.01	<0.001	0.05
Ammonia N, % of CP	1.1	0.3	0.6	0.8	0.9	1.3	0.3	0.3	0.11	<0.001	0.07	0.14
Zein, % of DM	4.9	4.8	5.1	4.6	5.3	4.5	4.9	4.8	0.41	0.88	0.29	0.37
Starch, % of DM	66.8	66.9	66.3	67.4	66.4	67.2	66.2	67.5	0.62	0.89	0.12	0.69
ivSD,^2^ % of starch	23.0	29.7	24.0	28.7	24.2^b^	21.8^b^	23.7^b^	35.7^a^	1.62	<0.001	0.01	<0.001

a–c Means within a row with different superscripts differ (*P* ≤ 0.05).

1 K = kernel effect (intact vs. broken); E = ensiling effect (0 vs. 30 d).

2 Ruminal in vitro starch digestibility at 7 h.

Although starch concentrations were unaffected by kernel form and ensiling (*P* > 0.10), an interaction was observed for ivSD (*P* < 0.001). The ivSD of kernels separated from silage stored for 0 or 30 d was not different when the kernels were intact. However, ivSD of separated kernels was greater when silage was stored for 30 d compared with 0 d when the kernels were broken. This finding is significant in and of itself because it suggests that an improvement in kernel starch digestibility with ensiling is limited to broken kernels. At least within the first 30 d of storage, our results indicate that protein solubilization and degradation by microorganisms, plant enzymes, and organic acids may be insufficient to improve starch digestibility when the endosperm is protected by an intact pericarp. Saylor et al. (2020) reported greater in situ starch degradation after 28 d of ensiling for finely ground compared with coarsely ground HMC and attributed this effect to physical (based on mean particle size) and chemical (based on soluble CP and ammonia N concentrations) characteristics of HMC. Greater proteolytic activity and concomitant improvements in soluble CP and ammonia N were associated with a more pronounced fermentation pattern (Saylor et al., 2020). Fermentation pattern was similar between kernel form treatments in the current study. Furthermore, the seemingly absent correlation between N fractions and starch digestibility in kernels separated from WPCS was unexpected. Although concentrations of soluble CP and ammonia N are correlated with starch digestibility in WPCS (Ferraretto et al., 2015) and HMC (Ferraretto et al., 2014), this correlation is stronger in HMC samples, presumably due to proteolysis occurring within the forage portion of WPCS. It is possible that some of the soluble CP and ammonia N measured in the separated corn kernels originated from proteins in the forage portion of WPCS and therefore had no connection to kernel starch digestibility.

The effect of kernel form and ensiling on the fermentation profile, nutrient composition, and N fractions of ensiled corn grain is shown in Table 3. An interaction between kernel form and ensiling was observed (*P* < 0.001) for pH. The pH of ensiled corn grain decreased from 0 to 30 d of storage for both intact and broken kernels, but the reduction in pH was greater when kernels were broken. A similar interaction was observed (*P* ≤ 0.01) for concentrations of lactic, acetic, and total acids. Concentrations of lactic and total acids increased as storage increased from 0 to 30 d for both intact and broken kernels. However, the increase in concentration was greater when kernels were broken. Concentrations of acetic acid were not different between 0 and 30 d of storage when kernels were intact, but they increased from 0 to 30 d when kernels were broken. Results from this experiment suggest enhanced fermentation in the ensiled corn grain when corn kernels were broken. This is likely related to a greater exposure of kernel sugars to microbial fermentation. Saylor et al. (2020) observed greater concentrations of lactic acid in finely ground HMC compared with coarsely ground HMC. Similarly, Baron et al. (1986) observed greater concentrations of lactic acid in ground compared with whole HMC ensiled at 36% moisture.

**Table 3:** Effect (means) of kernel form and ensiling on the fermentation profile, nutrient composition, and N fractions of ensiled corn grain

Item^1^	Kernel		Storage length		Intact		Broken		SEM	*P*-value^2^		
	Intact	Broken	0 d	30 d	0 d	30 d	0 d	30 d		K	E	K × E
pH	6.0	5.6	6.6	5.0	6.6^a^	5.4^b^	6.6^a^	4.5^c^	0.13	0.01	<0.001	<0.001
Lactic acid, % of DM	0.6	1.1	0.02	1.7	0.02^c^	1.3^b^	0.02^c^	2.1^a^	0.13	0.01	<0.001	0.01
Acetic acid, % of DM	0.04	0.2	0.04	0.2	0.04^b^	0.04^b^	0.04^b^	0.4^a^	0.04	<0.001	<0.001	<0.001
Total acids, % of DM	0.9	1.5	0.3	2.1	0.3^c^	1.5^b^	0.3^c^	2.7^a^	0.12	<0.001	<0.001	<0.001
DM, % as-fed	62.2	61.5	62.0	61.7	62.3	62.0	61.7	61.4	0.23	0.02	0.16	0.86
CP, % of DM	10.8	10.7	10.5	10.9	10.6	10.9	10.4	10.9	0.07	0.34	<0.001	0.28
Soluble CP, % of CP	21.2	26.0	19.4	27.8	19.5^b^	23.0^b^	19.2^b^	32.7^a^	1.00	<0.001	<0.001	<0.001
Ammonia N, % of CP	6.3	10.9	4.4	12.9	4.4^c^	8.3^b^	4.4^c^	17.5^a^	0.21	<0.001	<0.001	<0.001
Zein, % of DM	5.0	4.6	4.7	4.9	4.9	5.1	4.5	4.6	0.16	0.01	0.35	0.84
WSC, % of DM	2.3	2.7	3.5	1.5	2.9^bc^	1.8^c^	4.2^a^	1.2^c^	0.28	0.23	<0.001	0.004
Starch, % of DM	65.8	68.3	65.8	68.4	65.6^b^	66.1^b^	65.9^b^	70.7^a^	0.74	0.01	0.004	0.01
ivSD, % of starch	26.9	35.0	33.1	28.8	29.9	23.8	36.2	33.9	1.24	<0.001	0.03	0.31

a–c Means within a row with different superscripts differ (*P* ≤ 0.05).

1 WSC = water-soluble carbohydrates; ivSD = ruminal in vitro starch digestibility at 7 h.

2 K = kernel effect (intact vs. broken); E = ensiling effect (0 vs. 30 d).

Broken ensiled corn grain had (*P* = 0.02) slightly lower concentrations of DM compared with intact corn grain. Ensiling, however, had no effect on DM concentrations (*P* > 0.10). Although kernel form had no effect on CP concentrations (*P* > 0.10), ensiling slightly increased (*P* < 0.001) concentrations of CP. Interactions between kernel form and ensiling were observed (*P* < 0.001) for concentrations of soluble CP and ammonia N in ensiled corn grain. Soluble CP concentrations increased from 0 to 30 d of storage when kernels were broken but remained similar for intact kernels. Similarly, ammonia N concentrations increased from 0 to 30 d of storage for both intact and broken kernels, but the increase in ammonia N with ensiling was of greater magnitude when the kernels were broken.

Saylor et al. (2020) observed greater ammonia N concentrations in finely ground HMC compared with coarsely ground HMC after 28 d of storage. Baron et al. (1986) observed greater concentrations of soluble N in ground compared with whole HMC ensiled for 30 d at 33% moisture. In our study, fermentation was more pronounced for broken kernels than for intact kernels, which could explain these effects on soluble CP and ammonia N. Furthermore, surface area available for protein degradation is increased when corn kernels are broken and the endosperm is exposed. Increased concentrations of soluble CP and ammonia N in ensiled corn grain are indicative of the degradation of zein proteins in the kernels (Hoffman et al., 2011). Our results support this premise as concentrations of zein proteins were reduced (*P* = 0.01) in broken kernels compared with intact kernels. No effect (*P* > 0.10) of ensiling was detected on zein protein concentrations, however.

An interaction between kernel form and ensiling was observed (*P* = 0.01) for starch concentrations. Concentrations of starch were similar between 0 and 30 d for intact kernels but greater at 30 d compared with 0 d for broken kernels. This response may be related to a more robust fermentation with broken kernels, and the method used to determine DM as heat during oven-drying may volatilize most fermentation end products. Broken kernels had greater ivSD (*P* < 0.001) compared with intact kernels. Unexpectedly, ivSD was greater (*P* = 0.03) at 0 d compared with 30 d of storage. Because the protein matrix surrounding starch granules is the primary inhibitor of starch degradation in the rumen, it is understandable that broken kernels, having experienced a greater degree of proteolysis during storage, would contain more digestible starch than intact kernels. Accordingly, starch digestibility typically increases in HMC with ensiling and prolonged fermentation (Ferraretto et al., 2014). It is uncertain why ivSD was reduced with ensiling in the present study. Although our soluble CP and ammonia N data indicate that considerable proteolysis occurred in ensiled corn grain from 0 to 30 d of storage, our data also suggest that zein proteins may not have been the primary proteolytic substrate. Zein protein concentrations were not affected (*P* > 0.10) by ensiling in this experiment. It is possible that degradation of other kernel proteins may have occurred during storage. The 4 main components of a corn kernel are the endosperm, germ, bran, and tip cap. Each component contains protein of varying types and amounts. The endosperm, the largest part of the kernel, contains 60% zein, 26% glutelin, and about 6% each of albumin and globulin proteins (Wilson, 1987). Identification of the proteolytic substrate responsible for increased concentrations of soluble CP and ammonia N in this experiment requires further investigation.

To our knowledge, this is the first experiment to process corn kernels independent of the forage portion, reconstitute the kernel and forage portions to produce WPCS, and then analyze the chemical composition, fermentation profile, and starch digestibility of the WPCS, kernels separated from WPCS, and ensiled corn grain. Overall, this study gives insight into the importance of kernel breakage for improving starch digestibility in corn silage through means other than a reduction in particle size and opens the door for continued investigation into the proteolytic activity that occurs in the silo.
